# The Role of Surveillance in the Management of Small Renal Masses

**DOI:** 10.1100/tsw.2007.157

**Published:** 2007-04-30

**Authors:** Alessandro Volpe

**Affiliations:** Division of Urology, San Luigi Hospital, University of Turin, Regione Gonzole, 10043 Orbassano (Turin), Italy

**Keywords:** surveillance, renal mass, kidney cancer, natural history, renal cell carcinoma

## Abstract

Incidentally detected, small renal masses (SRMs) have been increasing significantly in recent years due to the widespread use of improved cross-sectional imaging. A significant number of incidental SRMs are diagnosed in elderly patients who are more likely to undergo imaging for other medical issues. The natural history of SRMs has not been historically well understood because most masses are surgically removed soon after diagnosis.

Several reports of surveillance of SRMs have been published in the last few years. When followed conservatively with serial imaging, SRMs have variable growth rates with an average of 0.28 cm/year, according to a recent meta-analysis. Larger series with longer follow-up are needed, but a significant number of small tumors seem to have an indolent behavior with a slow growth rate and a limited tendency to progress. The standard of care for enhancing SRMs is surgery. Up to one-third of surgically removed, <4-cm tumors are histologically benign. The outcomes of current surgical treatment of histologically confirmed, <4-cm, renal cell carcinomas are excellent, but this has not led to a decrease in mortality. Based on these considerations and on the available data on the natural history of SRMs, it seems reasonable to consider that we may be overtreating these lesions. This is especially true for elderly or unfit patients who have a decreased life expectancy. In these selected patients and in patients who refuse active treatment, it seems reasonable to propose an initial period of active surveillance for incidental SRMs, with delayed intervention for those tumors that will exhibit fast growth during follow-up. Percutaneous needle biopsies of renal tumors can be safely performed with the use of modern techniques and have the potential to characterize SRMs at histologically diagnosis, thereby allowing a better selection of the conservative or active treatment that is best suited for each individual patient.

Malignant tumors of the kidney cause about 2% of cancer incidence and mortality in the U.S., with an estimated 38,890 new cases and 12,840 deaths in 2006[1]. Since 1950, there has been a 126% increase in the incidence of renal cell carcinoma (RCC) in the U.S.[2]. A raising trend has been observed worldwide and is due, in part, to the widespread use of new and improved noninvasive abdominal imaging modalities, such as ultrasonography (US), computed tomography (CT), and magnetic resonance imaging

(MRI)[2,3,4,5,6,7,8,9,10,11,12,13]. The number of abdominal radiological exams has been increasing steadily in the last 2 decades and almost doubled between 1986 and 1994[5].

The incidence of RCC has increased in all age groups and in all clinical stages, but the greatest increase has been observed in localized tumors, which increased by 3.7%/year from 1973 to 1998[5,6]. Most RCCs are now incidentally detected by imaging as small renal masses (SRMs) in asymptomatic patients, while in the early 1970s, the incidental detection rate was only 7 to 13% and most renal tumors were diagnosed because of symptoms, such as flank pain and hematuria[14,15,16,17,18,19,20,21,22]. Tumor size at diagnosis has also substantially decreased over time. Series from the Memorial Sloan-Kettering Cancer Center and the Mayo Clinic report that the mean size of resected renal tumors has dropped from 7.8 to 5.3 cm from 1989 to 1998, and that there was a 32% decrease in mean tumor size at the time of diagnosis, respectively[23,24].

The incidentally detected lesions are, on average, smaller and present at an earlier stage than those detected in symptomatic patients[6,10,12,13,15,18,19,24,25,26,27,28,29,30,31]. Tsui et al. reviewed the records of 633 consecutive patients who underwent surgical treatment for RCC at UCLA between 1987 and 1998. Stage I lesions were discovered in 62.1% of patients with incidental RCC and 23% with symptomatic RCC ($p = 0.001$). Mean tumor size was 5.1 vs. 7.3 cm in incidental vs. symptomatic cases ($p < 0.05$)[13]. Patard et al. evaluated a series of 400 renal tumors and observed significantly smaller neoplasms in the incidentally detected group (5.7 vs. 8.7 cm; $p < 0.001$)[26].

A significant number of small asymptomatic tumors are benign; 32 and 33.6% of renal tumors that have been removed by laparoscopic partial nephrectomy at the Cleveland Clinic and at the Johns Hopkins Medical Institution, respectively, were found to be benign at pathology[32,33]. Frank et al. recently reviewed the pathology of 2,935 renal tumors removed at the Mayo Clinic and observed that, as tumor size decreases, there is a significant increase in the likelihood of having a benign tumor, a papillary compared to a clear cell histology, and a low-grade compared to a high-grade malignancy. In their experience, 30% of <4-cm, surgically removed tumors were benign at pathology and over 87% of those that were clear cell RCC were low-grade tumors[34].

Finally, several authors reported that small, incidentally detected tumors are characterized by better survival outcomes[10,13,15,20,22,26,28,35,36]. The first evidence of an association between tumor size and prognosis was reported by Bell, who noted an increased rate of metastases in patients found at postmortem to have RCCs >3 cm[37,38].

## NATURAL HISTORY OF SMALL RENAL MASSES

Small renal neoplasms are generally removed soon after diagnosis. Therefore, their natural history has been poorly understood historically.

In the landmark report of watchful waiting of kidney tumors, Bosniak et al. retrospectively reviewed the imaging of 40 incidentally detected, <3.5-cm, renal masses that had been followed for a mean of 3.25 years. Twenty six tumors were eventually removed after an average of 3.8 years and 84.6% of them were histologically RCCs. Variable tumor growth behaviors were observed and the overall mean linear growth rate was 0.36 cm/year (0–1.1 cm/year). Nineteen tumors grew at lesser than 0.35 cm/year and no patient developed metastatic disease[39,40,41].

In the first prospective study of surveillance of renal tumors at the University Health Network of Toronto, 32 incidentally diagnosed, <4-cm, renal masses were followed expectantly because the patients were elderly or unfit for surgery. Twenty five tumors were solid and seven complex cystic (four Bosniak III and three Bosniak IV). The patients were prospectively followed with serial abdominal imaging for a mean of 27.9 months (5.3–143) and each mass had at least three follow-up measurements. Tumor volume, in addition to single and bidimensional diameters, was calculated from each follow-up image or report. Nine masses in eight patients were surgically removed after an average of 38 months of follow-up because of the surgeon's concern or the patient's anxiety that the tumor was enlarging. All tumors were clear cell RCCs, except one that was an oncocytoma. The overall average growth rate, considered the cube root of the volume, was 0.1 cm/year and was not associated with either initial size ($p$ = 0.28) or mass type ($p$ = 0.41) (Fig. 1). Seven masses (22%) reached 4 cm in diameter after 12–85 months of follow-up. Eight (25%) doubled their volumes within 12 months. Overall, eleven (34%) fulfilled one of these two criteria of rapid growth. No patient progressed to metastatic disease, while two patients died of unrelated causes[42].

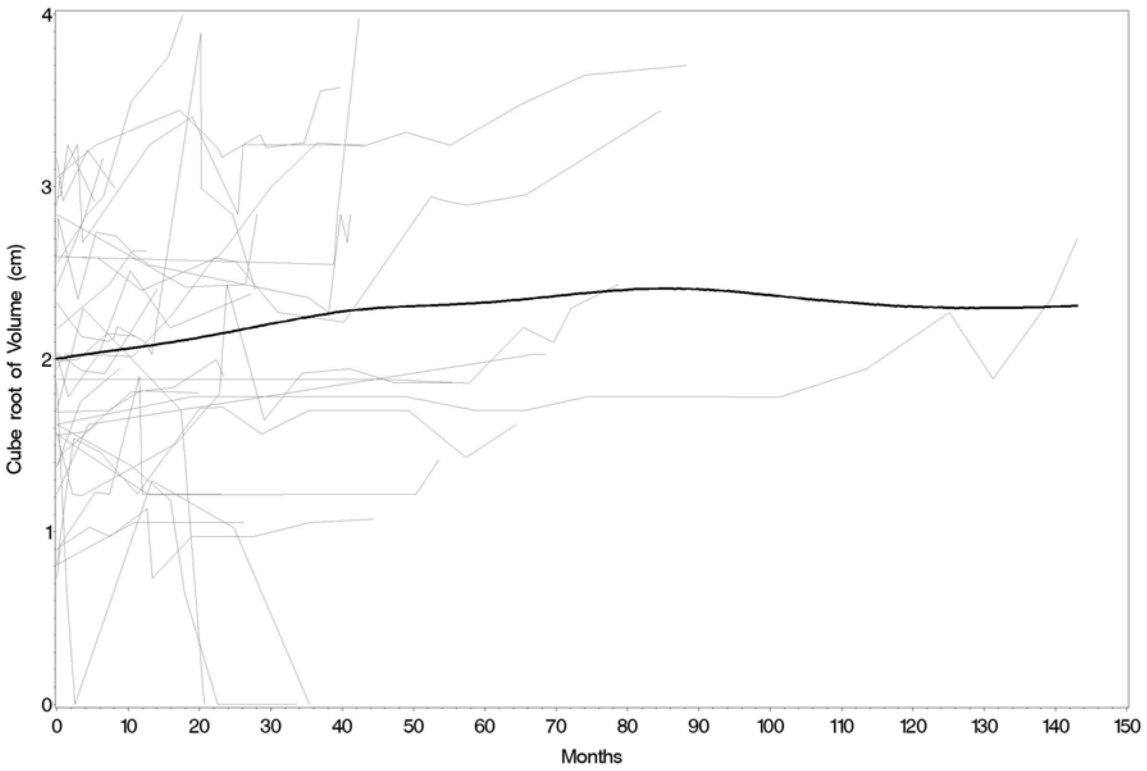

**FIGURE 1.** Individual observed patterns of tumor growth rate over time of 32 SRMs managed by active surveillance. A summary curve indicating the average growth rate is superimposed. (From Volpe et al.[42], with permission.)

A similar experience has been reported by Kassouf et al., who serially imaged 24 patients with SRMs. Most of the tumors did not demonstrate significant growth during the surveillance period. The mean growth rate of the five fast-growing tumors was 0.49 cm/year or 7.3 cc/year. The four tumors that were removed during the follow-up were all histologically RCC (three clear cell and one papillary type). No metastasis was documented[43].

Other experiences of watchful waiting of SRMs have been published in the last few years with similar results[44,45,46] (see Table 1). Chawla et al. recently carried out a meta-analysis of the available studies on this topic; 234 renal masses from eight different series, with a mean size at presentation of 2.6 cm, were included in the analysis. With a mean follow-up of 34 months, mean tumor growth rate was 0.28 cm yearly. Pathological confirmation was available in 46% of the cases and 92% of the masses were confirmed as RCC variants. Lesion size at presentation did not predict the overall growth rate ($p$ = 0.46)[47].

Interestingly, Lamb et al. observed a slow growth rate also in larger renal tumors. They followed a series of 36 renal masses with an average size of 7.2 cm at diagnosis, in patients who were considered unsuitable for surgery or were unwilling to have surgery. Two-thirds of the masses were biopsied and the diagnosis of RCC was confirmed in all cases except one. The authors observed a growth rate of 0.0–1.76 cm/year, with 55% of patients showing no increase in tumor size[48].

At present, progression to metastatic disease has been described for only three renal neoplasms managed by surveillance. However, the first tumor was already >8 cm in diameter at diagnosis, the second metastasized after growing to >8 cm during follow-up, and the third spread after more than 10 years of observation[46,47,48].

**TABLE 1**
**Outcomes of Studies of Active Surveillance for SRMs**

| Study | Number of Masses | Average Tumor Sze (cm) | Average Follow–Up (months) | Average Tumor Growth (cm/year) | Masses Eventually Surgically Removed (%) | Histologically Confirmed RCC (%) |
|---|---|---|---|---|---|---|
| Bosniak et al.[40] | 40 | 1.73 | 39 | 0.36 | 26 (65%) | 22/26 (85%) |
| Volpe et al.[42] | 32 | 2.48 | 27.9 | 0.1 | 9 (28%) | 8/9 (89%) |
| Kassouf et al.[43] | 24 | 3.27 | 31.6 | 0.09 | 4 (17%) | 4/4 (100%) |
| Kato et al.[44] | 18 | 1.98 | 22.5 | 0.42 | 18 (100%) | 18/18 (100%) |
| Wehle et al.[45] | 29 | 1.83 | 32 | 0.12 | 4 (14%) | 3/4 (75%) |
| Sowery and Siemens[46] | 22 | 4.08 | 26 | 0.86 | 2 (9%) | 2/2 (100%) |
| Chawla et al.[47] | 61 | 2.97 | 36 | 0.20 | 21 (34%) | 17/21 (81%) |

One of the limitations in interpreting these studies is the lack of pathological data on the tumors that remain on surveillance. However, since the vast majority of tumors that have been removed after a period of watchful waiting were histologically RCC and the same radiographic criteria were used to assess all masses, a large proportion of the remaining lesions should also be histologically malignant.

The studies on active surveillance that have been published to date are mostly retrospective, have a relatively short follow-up, and include a limited number of patients. However, their results are consistent and clearly suggest that a large number of incidentally detected SRMs have a slow growth rate and an indolent clinical behavior if managed conservatively.

## ACTIVE SURVEILLANCE OF SMALL RENAL MASSES

The standard of care for small, localized, renal neoplasms is either radical or partial nephrectomy. Nephron-sparing surgery, originally proposed for patients with a solitary kidney, impaired renal function, or bilateral tumors, is becoming the gold standard for smaller RCCs, given its comparable cancer control rate and reduced impact on renal function. Laparoscopic partial nephrectomy is a challenging technique, but it is already preferred to open partial nephrectomy in centers with advanced laparoscopic expertise.

The current management of SRMs yields excellent results. In the Mayo Clinic experience with surgical treatment of RCC, the 5-year cancer-specific survival for pT1a tumors is 97%[49]. In an international, multicenter study of 1,454 patients, Patard et al. reported a 5-year cancer-specific survival approaching 97% for pT1a tumors after nephron-sparing surgery[50]. In the Cleveland Clinic experience with laparoscopic partial nephrectomy, cancer-specific survival was 100% at a median follow-up of 42 months, with no local or port-site recurrence[32].

Morbidity from nephrectomy has decreased with improved techniques, but it is still significant and is reported to occur in 11 to 40% of cases in recent series[24,51,52,53,54]. Furthermore, most incidental tumors are detected in the elderly, who are more likely to undergo radiological examinations for other medical issues. These patients frequently have significant comorbidities and have a higher risk of perioperative mortality and morbidity.

Despite the significant increase in the diagnosis of localized neoplasms and the excellent outcomes of surgical treatment of SRMs, mortality of RCC has not decreased in the last few years and, in fact, it is slowly increasing. This probably implies that many small, incidental renal tumors have a long natural history and limited capacity to progress, while most RCCs that actually lead to death still present with symptomatic, locally advanced, or metastatic disease. This argument is supported by autopsy reports that shows that 67 to 74% of RCCs remained undetected until death before the widespread use of imaging and only 8.9 to 20% of undiagnosed RCCs were eventually responsible for the patient's death[3,55,56]

Based on these observations and on the analysis of the emerging data on the natural history of SRMs, it appears we may be overtreating these lesions and the current practice of immediate surgery for all newly diagnosed small renal tumors may have to be re-examined. In fact, it seems that a significant number of small, incidentally discovered, renal neoplasms are not histologically malignant or have an indolent clinical behavior. Therefore, they may not be an immediate threat to the patient's life. The risks of surgical treatment are only acceptable if the patient's life expectancy is longer than the time the tumor will take to progress. A recent study reviewed a series of 2,570 radical nephrectomies and indicated that approximately 5, 10, and 20% of patients who undergo surgery for RCC succumb to other cause mortality at 1, 10, and 20 years of follow-up[57]. In the Cleveland Clinic experience, 14% of patients died of other causes with a mean follow-up of 42 months[32].

A period of initial observation with delayed surgical treatment reserved for those tumors that exhibit a fast growth during follow-up, i.e., those that have rapid doubling times or whose volumes or bidimensional diameters reach a threshold we consider to be at risk for progression, may be appropriate in patients who are elderly or infirm. This is the concept of active surveillance. An upper limit of 3–4 cm in diameter and a volume doubling time >1 year are commonly used to identify renal masses that are at low risk of developing metastases and have a better survival rate[37,38,40,42,58,59,60,61]. We need further experience to propose definitive thresholds for treatment, but these appear to be reasonable limits for size and growth rate until which surveillance might be considered before suggesting therapeutic intervention in selected patients. There are reports that judicious delayed surgery does not appear to adversely impact clinical and pathological outcomes, even if the tumors are high grade[62,63]. However, large multicenter studies with long follow-up are needed to confirm the safety of active surveillance. At the present time, in the absence of effective treatment for metastatic disease, this strategy should not be recommended for young and fit patients.

The optimal follow-up schedule for patients in active surveillance has not been defined. At the University Health Network in Toronto, a triphasic, abdominal CT scan is performed at 3-month intervals for 1 year, then every 6 months to 3 years, and yearly thereafter if there is minimal or no growth. Monitoring SRMs is labor intensive and requires good patient compliance and careful organization.

The risk of measurement error at imaging is a concern in the conservative management of patients with small renal tumors. However, several authors reported reproducible and accurate tumor volume measurements by the use of CT scan and MRI[64,65,66,67]. A higher degree of inter- and intraobserver variability in measurements seems to occur with the use of US[68,69]. Masses with cystic components represent a special problem because tumor growth rate can be easily either overestimated or underestimated if the volume of cystic fluid grows at a different rate than the tumor cell volume.

Percutaneous needle biopsy is technically feasible today in an outpatient setting and can provide useful material for diagnosis in over 90% of cases in centers with expertise[70,71,72]. With the use of modern techniques, the risk of significant bleeding or needle track implantation is extremely rare. Significant grade heterogeneity is not very common in small tumors so that biopsies are likely to provide tissue that is representative of the entire tumor in the majority of cases. The histological characterization of SRMs with percutaneous biopsies has the potential to allow a better selection of patients for active surveillance. In fact, biopsies can identify the few small and high-grade tumors that should not be followed conservatively, but removed surgically up front. Also, a benign biopsy can dictate a less-intensive follow-up schedule, especially in elderly and frail patients.

A future goal is to go beyond classical histology and to identify genetic and molecular markers (members of the hypoxia-inducible/angiogenesis pathway, proliferation/apoptosis markers, adhesion molecules, etc.) that can reliably predict the clinical behavior of RCCs at needle biopsy. The high-throughput gene microarrays technology has the potential to provide significant prognostic information. In fact, there is increasing evidence that gene expression profiles can reveal histological subtypes and clinical outcomes of RCC[73]. Good-quality microarrays can be obtained with the amplification of the RNA from the small amount of tissue from needle biopsies.

In summary, the measurement of tumor growth rate seems to be helpful for initial conservative management of patients with incidentally diagnosed, small renal tumors. With a judicious use of delayed intervention for tumors with a fast growth rate, the risk of progression to metastatic disease appears to be little. However, longer follow-up is needed to confirm these observations. At the present time, active surveillance of SRMs should be considered only for elderly patients, patients with significant comorbidity, and patients who refuse active treatment. Needle biopsies can play a significant role in selecting those patients that are better candidates for this approach. Finally, the discovery of reliable genetic and molecular prognostic markers are needed in order to better differentiate small renal tumors with different aggressiveness and metastatic potential, thereby enabling the urologist to choose the most suitable conservative or active treatment for each patient.

## ACKNOWLEDGMENTS:

This review originates from the clinical and scientific experience with active surveillance of SRMs I developed during a 2-year urologic oncology fellowship at the University of Toronto, Ontario, Canada. A special acknowledgment goes to Dr. Michael A.S. Jewett, who has been an outstanding surgical and scientific teacher, and led me in the clinical research in this field.
